# ICD-11 Classification of Pediatric Chronic Pain Referrals in Ireland, with Secondary Analysis of Primary vs Secondary Pain Conditions

**DOI:** 10.1093/pm/pnab116

**Published:** 2021-03-26

**Authors:** Eveline Matthews, Geraldine Murray, Kevin McCarthy

**Affiliations:** 1 Children’s Health Ireland at Crumlin, Dublin, Ireland; 2 Children’s Health Ireland at Temple Street, Dublin, Ireland; 3 Department of Paediatrics, Trinity College Dublin, Dublin, Ireland

**Keywords:** International Classification of Diseases, Pediatric Pain, Chronic Pain, Primary Pain, Pain Catastrophizing, Diagnostic Uncertainty

## Abstract

**Objective:**

To classify pediatric chronic pain referrals in Ireland according to the classification system of the 11^th^ version of the *International Classification of Diseases* (ICD-11). In addition, differences between primary and secondary pain groups were assessed.

**Methods:**

Retrospective review of complex pain assessment forms completed at the time of initial attendance at pediatric chronic pain clinics in Dublin, Ireland. Patients were classified as having a chronic primary (CPP) or chronic secondary (CSP) pain condition as per ICD-11 classification. Secondary analysis of between-group and within-group differences between primary and secondary pain conditions was undertaken.

**Results:**

Of 285 patients coded, 123 patients were designated as having a CPP condition (77% of whom were assigned an adjunct parent code) and 162 patients as having a CSP condition (61% of whom were assigned an adjunct parent code). Between-group comparisons found that the lowest reported pain scores were higher in CPP than in CSP conditions. There were stronger correlations between parental pain catastrophizing and pain intensity, school attendance, and pain interference with social activities in the CSP group than in the CPP group.

**Conclusions:**

The majority of children with both CPP and CSP were assigned multiple parent codes. There appears to be a gradient in the differences in biopsychosocial profile between CPP and CSP conditions. Additional field testing of the ICD-11 classification in pediatric chronic pain will be required.

## Introduction

Pediatric chronic pain is best managed within a biopsychosocial formulation [1]. Such a formulation should posit that biological, psychological, and social factors interact dynamically to cause and maintain pain. Successful treatment requires a multidisciplinary approach addressing each of these domains. However, the management of pediatric chronic pain conditions is not uniform or generic. Each domain may be emphasized differently, depending on the individual child, the pain etiology, and the current evidence base. For example, if multidisciplinary management is framed as a combination of physical/physiological, pharmacological, and psychological therapeutic modalities [2], the current evidence bases for different conditions place greater emphasis on different modalities, e.g., physical therapies for pediatric complex regional pain syndrome (CRPS) [3], psychological therapies for functional abdominal pain [4], and pharmacological therapies for painful sickle cell crises [5].

Biopsychosocial formulations are at the junction between personalized and evidence-based medicine. A formulation is a map to guide treatment. However, that map must be revised as children grow and develop, as they respond to therapeutic interventions, and as new clinical evidence emerges. The development of evidence bases requires groups of similar patients from which to make reasonable recommendations about treatment and to forecast outcome. This is becoming a more compelling requirement in pediatric chronic pain. A retrospective audit of a national database of inpatient admissions in the United States found an 831% increase in the number of patients with a chronic pain diagnosis between 2004 and 2010 [6]. Such dramatic increases in prevalence are typically driven by increased detection or changes in diagnostic criteria and, in the case of chronic pain, an increasing acceptance of and willingness to diagnose primary pain disorders. The clinical terminology and understanding of primary pain disorders are still evolving. However, a core feature of these disorders is ongoing pain, distress, and disability in the absence of a known biochemical or structural cause [7]. Previously, these pain entities may have been labeled as “functional” [8] or have had multiple organ-specific synonyms, such as “pain amplification syndrome,” “juvenile fibromyalgia,” or “chronic widespread pain” in the context of chronic musculoskeletal pain [9].

The addition of a chronic primary pain category was one of several major changes to the 11^th^ version of the *International Classification of Diseases* (ICD), which was made in conjunction with an International Association for the Study of Pain (IASP) Task Force [10]. The ICD-11 now has seven parent groupings for common chronic pain conditions, including chronic primary pain (CPP) and six chronic secondary pain (CSP) categories: chronic cancer-related pain, chronic posttraumatic or postsurgical pain, chronic neuropathic pain, chronic secondary headache or orofacial pain, chronic secondary visceral pain, and chronic secondary musculoskeletal pain. CPP is a new phenomenological entity of which the core feature is chronic pain “that cannot be better explained by another chronic pain condition” [10]. Just as multiple pain mechanisms (i.e., nociceptive, nociplastic, and neuropathic) may coexist and contribute to a person’s pain state, it is also accepted that patients may have more than one parent code (e.g., cancer pain and postsurgical pain, or primary pain and headache). However, what is not clear is how to determine the primacy of one code over another; for example, some patients with nociplastic pain etiologies might simultaneously be “both” and “neither,” depending on how a clinician interprets the taxonomy. An additional consideration in pediatric chronic pain is that adult diagnostic criteria may be an imperfect fit. For example, a child with suspected CRPS who does not meet the Budapest Criteria [11] might still be managed as “CRPS probable” in the same way as a child who does meet the criteria, as pediatric CRPS can have a spectrum of presentations and trajectories [2].

The primary objective of this retrospective study was to classify the pediatric chronic pain referrals to our service. Additional outcomes of secondary interest included any differences between primary and secondary pain groups in other variables, such as pain intensity and duration, school attendance, and potential psychosocial predisposing risk factors for chronic pain, such as parental pain catastrophizing, birth order, and social deprivation.

## Methods

### Participants

After institutional ethical approval, we performed a retrospective, cross-sectional analysis of referrals to a national pediatric complex pain service at Children’s Health Ireland, which is based across two tertiary pediatric hospitals in Dublin, Ireland.

### Complex Pain Assessment Form

Before their first clinical appointment with the pain service, children and their parents complete a complex pain assessment form, which records pain intensity and duration and parental estimates of children’s pain intensity and coping ability, each of which are recorded on an 11-point numerical rating scale (NRS). Additional factors recorded included identifiable triggering event, i.e., surgery, trauma, illness, or “other,” anatomic location of pain, and pain characteristics. The impact of pain on school attendance is recorded by self-reported number of days missed and percentage school attendance. Pain interference with social activities is recorded on a self-reported 11-point NRS (0= “no interference” to 10=“completely interferes”). We also administer the Pain Catastrophizing Scale–Parent (PCS-P) [12], as in the context of pediatric pain, high levels of parental pain catastrophizing have been linked to poor outcomes for children with pain, likely through an influence on child pain catastrophizing [13]. Age, sex, and birth order are recorded as part of general demographic data.

### CRPS Referrals

In our model of care, CRPS referrals are fast-tracked as a clinical priority. For these patients, we administer a checklist of the Budapest Criteria at the time of initial review and record whether or not they meet the criteria. Patients who do not meet the criteria may still be labeled “CRPS probable” and receive the same management. These data are recorded only for patients queried as possible CRPS by a referrer, not where CRPS was diagnosed by our service after the initial review. This is part of ongoing quality-control measures for monitoring the integrity of the fast-track process.

### Social Deprivation Index

Social deprivation was assessed with the Pobal HP Deprivation Index [14]. This index provides a method of analyzing comparative affluence and deprivation by geographic area derived from data from the 2016 National Census of Ireland. The Relative Index Scores are specific to that census wave and are rescaled to have a mean of zero and a standard deviation (SD) of ten. The scores approximately follow a normal distribution in eight categories, from “extremely affluent” (>3 SD above the mean) to “extremely disadvantaged” (>3 SD below the mean). The Relative Index Score and corresponding deprivation category for each participant were determined by entering their postal code into the Pobal online portal.

### Classification Strategy

The ICD-11 classification of chronic pain, unspecified, acknowledges seven categories under which chronic pain can be classified and coded [10]. CPP is a disease in its own right, where no other cause can be found for pain that persists for longer than 3 months and is associated with emotional distress and functional disability [7]. The other six categories, chronic cancer-related pain, chronic neuropathic pain, chronic secondary visceral pain, chronic posttraumatic or postsurgical pain, chronic secondary headache or orofacial pain, and chronic secondary musculoskeletal pain, where pain is recognized as a symptom of a disease, are termed CSP conditions [15]. There is further hierarchical subcategorization from the seven main parent codes, as “parent/child” entities. Multiple parent coding is also possible, with one designated as the “primary” parent code [10].

The first wave in the classification process was to use the information contained within the assessment form to determine whether the main parent code for a presentation was a primary or secondary pain entity. The main determining criterion was the definition of CPP as entities that “cannot be better explained by another chronic pain condition.” If uncertainty existed, the assessor was advised to assign an additional adjunct parent code. The second wave was to reach agreement between assessors on the main parent code and to subcategorize further, i.e., CPP as widespread CPP, localized CPP, or other CPP (including CRPS). For triggering events, a single, minor triggering event without evidence of ongoing inflammation or permanent structural change that led to chronic pain was classified as CPP, i.e., CRPS after a minor, completely resolved, soft tissue injury. Patients reporting multiple, repeated, or major triggering events or episodes of inflammation or patients with a progressive underlying condition were classified as having CSP, i.e., patients with hypermobility or scoliosis and probable central sensitization. Multiple parenting, or retention of a second adjunct parent code, occurred for one of three reasons: 1) The pain entity definitively spanned two parent categories (i.e., CRPS, which is both primary and neuropathic); 2) the pain entity spanned two parent categories, one definitive and one probable/possible (i.e., several of the patients with coexisting peripheral and central sensitization); or 3) the pain entity was not definitively in any one category (i.e., presentations that may be transitional at the time of initial review and may be easier to categorize at a future date after longitudinal follow-up). The final decision on parent codes required the agreement of an assessor who was directly involved in that individual patient’s clinical evaluation.

### Analytic Strategy

We compared differences between those who had CPP and those who had CSP as their main parent code to examine whether patients classified as having primary or secondary conditions had different profiles in terms of baseline demographics, potential pain risk factors, pain intensity scores, or outcomes such as school attendance. For this between-group analysis, we used a Mann-Whitney test for continuous variables and a chi-squared test for categorical variables. Where there were significant differences between groups or significant correlations within groups, we re-analyzed those variables for four “main code–adjunct code” combinations: “CPP–no adjunct,” “CPP-adjunct,” “CSP-adjunct,” and “CSP–no adjunct.” We used a Kruskal-Wallis one-way analysis of variance with Dunn’s multiple-comparisons test for pairwise post hoc comparisons. Data are presented as median and interquartile range (IQR) unless otherwise stated. Significance was set at α = 0.05. To examine relationships between variables within groups, Spearman correlation matrices were calculated. This was performed for CPP and CSP main codes and for the CPP–no adjunct and CSP–no adjunct subgroups. As an additional measure to informally assess the validity of our classification strategy, we also recorded the number of CRPS referrals that did and did not meet the Budapest Criteria that were classified as CPP or CSP with or without an adjunct code. We did not formally statistically test predictive values, as pediatric patients may still have CRPS despite not meeting the Budapest Criteria; therefore these data are presented for descriptive purposes only.

## Results

### Participants

We retrieved assessment sheets on 289 patients referred to the pediatric chronic pain service between February 2016 and November 2019. Four forms were incomplete and had insufficient information to assign a category, so they were excluded from subsequent analysis. Patients (33% male) had a median age of 13 years (IQR 11–15 years) and had a median duration of pain of 24 months (IQR 7.5–48.5 months). We identified 30 CRPS referrals where the Budapest Criteria were recorded and available; 19 patients met the criteria, and 11 patients did not.

### Main and Adjunct Parent ICD-11 Codes

**Figure 1.**
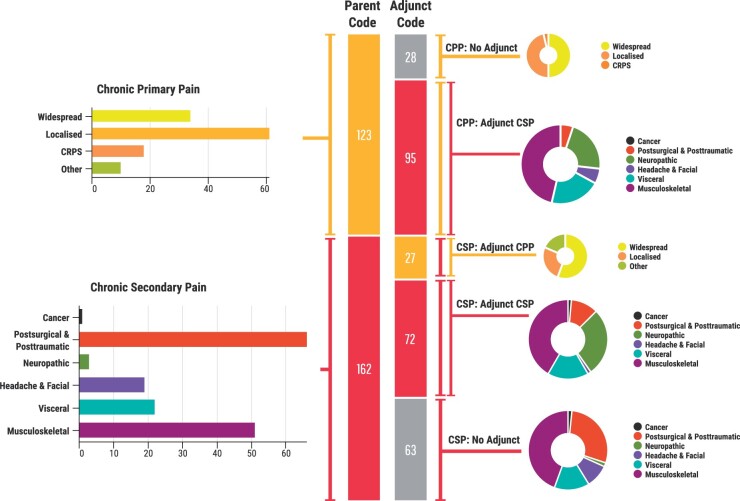
ICD-11 classification of pediatric chronic pain referrals. Patients (n=285) were assigned ICD-11 parent codes. Of those, 123 patients were assigned CPP as a main parent code, and 162 patients were assigned a CSP as a main code: chronic cancer pain (n=1), chronic postsurgical and posttraumatic pain (n=66), chronic neuropathic pain (n=30), chronic headache and facial pain (n= 19), chronic visceral pain (n=22), or chronic musculoskeletal pain (n=51). In patients with primary pain as the main parent code, 95/123 were assigned an adjunct parent code. In patients with secondary pain as a main parent code, 99/162 were assigned an adjunct parent code.

**Figure 2.**
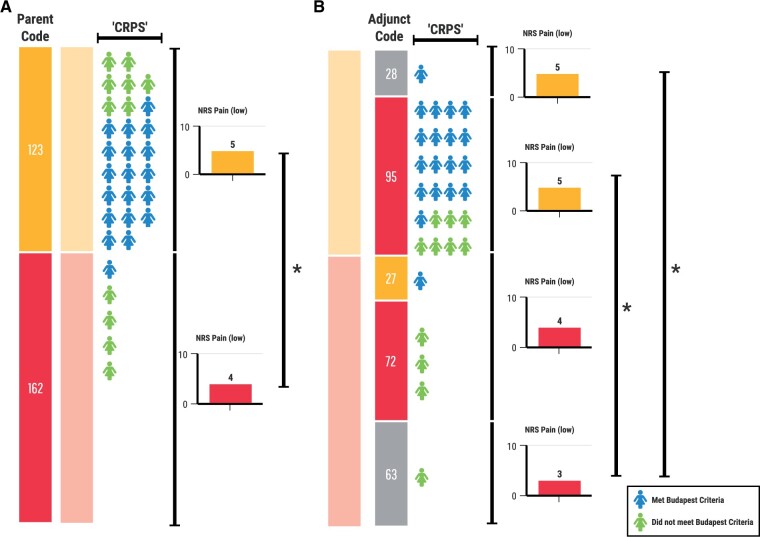
CRPS referrals and between-group differences. **(A)** Of the 30 CRPS referrals, 25 were classified as CPP (18/19 met the Budapest Criteria and 7/11 did not) and 5 as CSP (1/19 met the Budapest criteria, 4/11 did not). Lowest reported pain scores were significantly different between CPP and CSP main parent code groups (NRS pain (low) of 5 vs 4; *P*=0.006). **(B)** Of the CRPS referrals classified as CPP, 24 received an adjunct code, most frequently chronic neuropathic pain (n=20). The difference in lowest reported pain remained between subgroups (Kruskal-Wallis 11.34, *P*=0.01), specifically between the “CSP–no adjunct” subgroup and the two CPP subgroups “CPP–no adjunct” (*P*=0.048) and “CPP-adjunct” (*P*=0.015), on post hoc testing with Dunn’s multiple comparison test. There is a small gradient in lowest pain scores from “CPP–no adjunct” (NRS 5) to “CSP–no adjunct” (NRS 3).

Of the 285 patients assigned a main parent code, 123 were assigned a CPP code, and 162 were assigned a CSP code. Of those with CPP as the main parent code, 95 (77%) were assigned an additional adjunct parent code, with musculoskeletal pain (n = 45), neuropathic pain (n = 21), and visceral pain (n = 20) being the more commonly assigned categories. Of those with a CSP main parent code, 99 (61%) were assigned an adjunct parent code, with musculoskeletal pain (n = 30), primary pain (n = 27), and neuropathic pain (n = 20) as the more commonly assigned adjunct parent codes. The breakdown of the main codes and the main code–adjunct code pairings are shown in Figure 1. Of the 30 CRPS referrals, 25 were classified as CPP (18 of the 19 who met the Budapest Criteria and 7 of the 11 who did not) and 5 as CSP (1 met the Budapest criteria, 4 did not) [Figure 2A]. Of the CRPS referrals, 22 received an adjunct parent code of chronic neuropathic pain: 20 of the 25 classified as CPP and 2 of the 5 classified as CSP.

### Between-Group: CPP vs CSP Main Codes

The demographic variables, pain intensity scores, and pain-related disability measures (school attendance and interference with social activities) and the parental measures of child’s pain, child’s coping ability, and parental pain catastrophizing (P-PCS) for patients assigned CPP and CSP main codes are listed in Table 1. There were no differences in age, birth order (firstborn vs other), triggering event (no identifiable event vs specific event), pain duration, or social deprivation index between the two groups. The percentage of males was significantly higher in the CSP group than in the CPP group: 39.1% vs 26.8% (χ^2^ = 4.71, degrees of freedom [df] = 1, *P* = 0.029). There was a significant difference in lowest reported pain between the CPP and CSP groups (5 vs 4; *P* = 0.006) but no difference in highest reported pain or “pain on average” between the two groups. In terms of measures of pain-related disability, there were no differences in school attendance or social interference between the two groups. There were no major differences in parental measures between the two groups. Parental estimates of child pain and child coping and total P-PCS scores were not significantly different. Parental scores on the magnification subscale of the P-PCS were higher in the secondary pain group (5 vs 4; *P* = 0.02).

**Table 1.** Between-group comparisons of primary and secondary pain groups

	Primary Pain	Secondary Pain	*P* Value
	N = 123*	N = 162*	
**Demographics**			
Age, years (median)	13 (n = 118)	14 (n = 153)	0.29^a^
Sex, male (%)	33/123 (26.8%)	63/161 (39.1%)	**0.029^b^**
Trigger			
None	40	40	0.14^b^ (none vs event)
Surgery	5	31	
Injury	30	44	
Illness	18	30	
Other	30	17	
Birth order			
First (%)	59 (50%)	75 (48%)	0.70^b^
Later	58	81	
Total	117	156	
Pain duration, months (median, IQR)	22 (6–168)	25 (9–308)	0.106^a^
Social Deprivation Index, (median)	4	5	0.245^a^
**Pain intensity, 0–10 NRS**			
Average (median, IQR)	7 (6–8)	7 (5–8)	0.113^a^
Highest (median, IQR)	9 (8–10)	9 (8–10)	0.320^a^
Lowest (median, IQR)	5 (3–6)	4 (2–6)	**0.006^a^**
**Pain-related disability**			
School			
Days missed (median)	15 (n = 76)	13 (n = 101)	0.30^a^
% Attendance (median)	75% (n = 35)	75% (n = 45)	0.61^a^
Social			
Interference: 0–10 NRS (median)	8 (n = 97)	7 (n = 131)	0.74^a^
**Parental measures**			
Child’s pain intensity			
Pain intensity: average 0–10 NRS (median, IQR)	7 (5–8)	7 (5–8)	0.70^a^
Child’s pain coping			
Pain coping ability: 0–10 NRS (median, IQR)	6 (4–7)	6 (4–8)	0.99^a^
PCS-P (median, IQR)			
Total	28 (20–36)	31 (21–39)	0.24^a^
Rumination	13 (10–15)	13 (11–15)	0.45^a^
Magnification	4 (2–6)	5 (3–8)	**0.02^a^**
Helplessness	12 (8–16.5)	13 (7.5–18)	0.49^a^

* Sample size for full group; for outcomes where data was not available for all participants, the number of participants (n=*x*) is included.

a Obtained using Mann-Whitney test.

b *P* values by chi-squared test.

Values in bold represent *P* <0.05. IQR=Interquartile Range.

### Between-Group: Main-Adjunct Code Subgroups

We retested the significantly different between-group variables, and only the lowest pain remained significantly different (Kruskal-Wallis 11.34, *P* = 0.01). On post hoc testing with Dunn’s multiple comparison test, the differences were between the two CPP subgroups (“CPP–no adjunct,” “CPP-adjunct”) and the “CSP–no adjunct” subgroup (Figure 2B).

### Within-Group Analyses

There were multiple weak correlations between various variables. These were not stable and varied between the main groups and subgroups. We report the correlation between parental pain catastrophizing (P-PCS) and child pain intensity NRS, parental estimate of child’s pain and coping NRS, school attendance, and social interference in Table 2. The correlation between P-PCS and child and parent pain NRS was stronger in the CSP main group and subgroup than in the CPP main group or subgroup. The full correlation matrices are supplied in the Supplementary Data.

**Table 2:** Within-group Spearman correlations of main groups and subgroups

	Parental Pain Catastrophizing Scale (Total)			
	CPP N= 123	CPP, No Adjunct Code N = 28	CSP N = 162	CSP, No Adjunct Code N = 63
NRS c-Pain: Average	0.200	0.278	0.336	0.264
	0.036	0.179	<0.001	0.070
NRS c-Pain: High	0.253	–0.257	0.247	0.244
	0.009	0.237	0.003	0.085
NRS c-Pain: Low	0.196	0.256	0.345	0.320
	0.050	0.239	<0.001	0.027
School Attendance (%)	–0.179	0.949	–0.389	–0.332
	0.311	0.167	0.012	0.165
NRS Social Interference	0.247	0.101	0.242	0.443
	0.017	0.680	0.008	0.004
NRS p-Pain: Mean	0.289	0.060	0.456	0.529
	0.002	0.776	<0.001	<0.001
NRS p-Coping	–0.265	–0.084	–0.182	–0.227
	0.006	0.696	0.031	0.106

c-Pain=children’s pain score; p-Pain=parent’s estimate of child’s pain; p-Coping=parent’s estimates of child’s coping ability. NRS=Numerical Rating Scale; CPP=Chronic Primary Pain; CSP=Chronic Secondary Pain.

## Discussion

These results show that there may be differences in the biopsychosocial profiles of primary vs secondary pain conditions in pediatric chronic pain. For several reasons, the majority of patients were assigned multiple parent codes. The subgroups generated by multiple coding revealed possible gradients across a spectrum, with patients who have unequivocally primary pain at one end and those who have unequivocally secondary pain at the other. Perhaps counterintuitively, parental pain catastrophizing may exert more influence in secondary than in primary pain conditions. This is the first reported categorization of a nationally representative cohort of pediatric chronic pain clinic referrals as per the ICD-11 categories.

Although this attempt at formal categorization into primary and secondary pain conditions is previously unreported, our findings build on and align with previously undertaken work. Two thirds of children with chronic secondary musculoskeletal pain attending a tertiary pediatric chronic pain centre reported a precipitating event or trigger for their pain, in descending order of frequency: injury, chronic disease, infection or illness, and surgery [16]. Although our intake form is perhaps less detailed, patients in our primary pain group are broadly similar in profile: 68% reported a definite precipitating event, in descending order: injury, “other,” illness, and finally, surgery. In the secondary pain group, 76% reported a definite trigger, with a different descending order: injury, surgery, illness, and “other.” This is unsurprising, given that the chronic posttraumatic or postsurgical parent code is in this group. The difference in the proportion of patients reporting no precipitating event or trigger was not significant between the primary and secondary groups.

Although not statistically significant between groups, the fact that 68% of primary pain patients report a definite precipitating event may be important when it comes to buy-in to the biopsychosocial formulation. This diagnostic uncertainty may lead children and parents to struggle with the provided diagnosis and thus may result in a desire to continue to search for alternative explanations, damaging trust in the treating clinicians [17]. This may also be exacerbated by a sudden shift in emphasis from a biomedical diagnostic or therapeutic treatment algorithm to a biopsychosocial explanation [2], where a secondary pain condition has previously been provided as an explanation or is used synonymously. A formulation must acknowledge the initial trigger and the distinction between, and uncoupling of, peripheral and central sensitization in what may mechanistically fit with a nociplastic etiology [18]. It has been our practice to use mechanistic descriptors and therapeutic metaphor in our formulations in preference to formal diagnostic codes [2]. Therefore, a “CRPS probable” would receive the same formulation as a patient who meets the Budapest Criteria for CRPS. This also influenced our use of multiple parent codes, the goal being to add an additional descriptive layer so that we may analyze therapeutic outcomes for currently undefined subgroups. Much of our pain education focuses on neuroplasticity and locus of control, emphasizing that engagement with a multidisciplinary management plan may render a diagnosis redundant as symptoms may change over time.

The relative contributions of peripheral and central sensitization to pain states in primary vs secondary pain conditions may account for our finding of a significant difference in lowest reported pain between the two groups. As ongoing central sensitization in the absence of ongoing tissue injury or inflammation is a conceptual cornerstone in primary pain, it is plausible that baseline pain intensity is maintained at a higher level. In contrast, in secondary pain conditions, pain is a symptom of disease and likely driven by peripheral sensitization (that is also possibly amplified by central sensitization); therefore, baseline or resting pain scores may be reduced by medical management of the peripheral inflammatory or structural process driving the sensitization.

Emerging clinical guidelines emphasize physical and psychological therapeutic modalities for primary pain disorders [19]. For both patients and clinicians, this may unintentionally carry the implication that psychosocial factors are directly causal in primary pain, which may lie in conflict with the beliefs of those who identify a specific trigger or precipitating event. Parental pain catastrophizing, a construct comprised of rumination, magnification, and helplessness, has been associated with children’s pain and pain-related disability in multiple studies, an effect that is likely mediated through child pain catastrophizing [12, 20–22]. Again, perhaps somewhat counterintuitively, in the secondary pain group, the scores for the magnification subscale were higher, and there were stronger correlations between parental catastrophizing and child pain scores, and with parental estimates of child pain and child coping, as well as a correlation with social deprivation index.

Social and family factors such as social deprivation and birth order have been implicated in some long-term health outcomes into adulthood [23, 24]. The social deprivation index was not significantly different between the primary and secondary pain groups and was associated with parental pain catastrophizing and parental coping estimates only in the secondary pain group. The percentage of firstborns vs later-borns was also the same in both groups. However, birth order was negatively correlated with patient’s age in the secondary pain group. More recent work has shown that although firstborns have worse health at birth and that this health disadvantage persists to the age of 7 years, it then disappears and becomes a health advantage in adolescence [25]. In contrast, later-born children are throughout childhood more likely to suffer an injury and have worse mental health and alcohol-related hospital admissions as young adults [26]. Our findings align with that general trend, in that patients in the secondary pain group were younger in age if later-born, and this correlation was even stronger in those with chronic posttraumatic or postsurgical pain as a main parent code (Supplementary Data). Injury is more common in later-borns, and it is speculated that this is due to differential postnatal parental investment.

Injury might also be a factor in the sex difference between groups and the greater proportion of males in the secondary pain group. Alternatively, as women are disproportionately affected by chronic pain conditions in adulthood, a greater proportion of female children with primary pain may reflect a sex ratio that is present across all age categories or emergent after the onset of puberty. This may be related to increased pain sensitivity due to endocrine differences and a greater number of painful events (i.e., menstruation), or gender biases in pain recognition and undertreatment of pain in females, either of which may lead to increased pain responses over time. The former is an example of nociplastic pathophysiology and the latter is an example of the Social Communication Model of pain in action [27].

The lack of a template for classifying pediatric patients was initially challenging. We used the published literature as guidance [7, 14, 25]. However, categorizing children with underlying congenital conditions, such as scoliosis or connective tissue disorders, who also had likely nociplastic pain etiologies, meant that 68% of our patients were assigned two parent codes. Sixteen percent of our patients with a secondary pain main parent code were assigned a primary pain as an adjunct parent code, which reflects this clinical conundrum of coding nociplastic etiologies, where peripheral structural or inflammatory processes coexist with presumed central sensitization. This is in contrast to pilot field testing in adult pain clinics [28], where 20% of patients were assigned dual codes, although this coding was performed on consecutive patients seen in clinic, presumably also including review appointments, whereas we were using our intake form at referral. We chose to use our complex pain assessment intake form as the source, rather than the medical records, as the information is recorded in a systematic manner and completed by children and their parents themselves. It is possible that the rate of dual parent codes would decrease with training and experience in ICD coding and when a longitudinal relationship has been established. It also may be that children with pain-related disability that is sufficiently significant to warrant referral to a pediatric chronic pain service are complex.

In a retrospective audit of a national database in the United States, comorbid diagnoses were common: 65% had a gastrointestinal diagnosis and 44% a comorbid mood disorder, with a mean of 10 diagnoses per patient [6]. The differences in lowest pain score, parental pain catastrophizing, and the category allocations of the CRPS referrals each appear to follow a gradient, with patients who unequivocally have CPP at one end and those who unequivocally have CSP at the other. We would infer that this is indirect evidence of the validity of our categorization process. Those in between, with different combinations of main and adjunct parent codes, may reflect diagnostic uncertainty or distinct subgroups that may behave differently from each other. Therefore, generic treatment pathways for “primary pain” or “secondary pain,” without additional phenotyping, may be inappropriate.

This is a retrospective, cross-sectional study; therefore, the results must be interpreted cautiously. However, our findings may be hypothesis generating and highlight the need to conduct pilot field testing of the ICD-11 in pediatric chronic pain clinics to determine what adaptations, if any, are needed to improve clinical utility and diagnostic confidence in its use. Once variability in assigning codes has been addressed and minimized, real-world differences between primary and secondary pain conditions may be examined further.

## Supplementary Data

Supplementary Data may be found online at http://painmedicine.oxfordjournals.org.

## Supplementary Material

pnab116_Supplementary_Data

## Notes

Funding sources: This research did not receive any specific grant from funding agencies in the public, commercial, or not-for-profit sectors.
